# Functional and clinical outcomes of delusional disorder and schizophrenia patients after first episode psychosis: a 4-year follow-up study

**DOI:** 10.1186/s12888-023-05175-z

**Published:** 2023-09-18

**Authors:** Christy Lai Ming Hui, Evie Wai Ting Chan, Priscilla Wing Man Hui, Tiffany Junchen Tao, Elise Chun Ning Ho, Bertha Sze Ting Lam, Sally Hiu Wah See, Yi Nam Suen, Wing Chung Chang, Sherry Kit Wa, Edwin Ho Ming Lee, Eric Yu Hai Chen

**Affiliations:** 1 Department of Psychiatry, School of Clinical Medicine, Li Ka Shing Faculty of Medicine, University of Hong Kong, Hong Kong, Hong Kong; 2 State Key Laboratory of Brain and Cognitive Sciences, University of Hong Kong, Hong Kong, Hong Kong

**Keywords:** Schizophrenia, Paranoid schizophrenia, Psychotic disorders, Psychopathology, Psychosocial functioning

## Abstract

**Background:**

Literature has typically associated delusional disorder with a poorer prognosis relative to schizophrenia, without considering the confounding effect of age despite the differential age of onset. This study therefore aims to investigate the diagnostic stability, clinical, functional, and neurocognitive differences of Chinese first-episode psychosis age-matched patients with delusional disorder and schizophrenia at four years.

**Methods:**

71 delusional disorder and 71 age-matched schizophrenia patients were followed up for four years after their initial episode. Their symptoms, insight in psychosis, side effects of medication, medication compliance, functioning, and neurocognitive performance were assessed at four years.

**Results:**

At four years, 65% of DD patients maintained the same diagnosis, while the rest shifted to SZ. Only those without a diagnostic shift were included in the analysis. Delusional disorder patients (*n* = 46) experienced greater general psychopathology and poorer insight, but better attitude towards medication than schizophrenia patients (*n* = 71). Social and occupational functioning, quality of life, and cognitive functioning, however, were similar in delusional disorder and schizophrenia patients.

**Conclusions:**

Results indicate that delusional disorder is less diagnostically stable than schizophrenia. Their outcomes in a Chinese population were largely similar at four years after removing the confounding age factor, implying that delusional disorder and schizophrenia may not be as distinct as previously thought.

**Supplementary Information:**

The online version contains supplementary material available at 10.1186/s12888-023-05175-z.

## Introduction

Delusions can be defined as strongly-held abnormal beliefs that are bizarre and impervious to evidence, and which may be categorised according to their contents [1]. In the past, delusional disorder (DD) was differentiated from schizophrenia (SZ) by the requirement of non-bizarre delusions without hallucinations [2–4]. However, the DSM-V [5] now allows for bizarre delusions or relevant non-prominent hallucinations to co-occur with delusion(s). With the boundaries becoming less distinct, it is important to clarify the differences between DD and SZ for diagnostic accuracy and the optimisation of clinical treatment and management of patients.

Previous research have suggested that compared to SZ, DD is associated with a greater predominance of jealousy and somatic delusions [6], little or non-existent hallucinations [7], and less cognitive and negative symptoms [8]. These findings generally indicate DD as a milder psychotic disorder than SZ, supporting the idea of a psychosis spectrum that may encompass DD, SZ, and schizoaffective disorder in escalating severity [8].

In terms of longitudinal stability, only 59% of DD patients maintained the same diagnosis at follow-up, with approximately one-third later diagnosed with SZ [9]. In fact, 93% of diagnostic changes within the SZ spectrum occurred towards SZ. Therefore, refining their longitudinal clinical characterisation and profiling may contribute towards the debate of a categorical or a dimensional descriptive approach, where DD may be better subsumed under SZ [10].

### Comparisons between DD and SZ cohorts

Few studies have compared DD with other psychoses due to recruitment difficulties. On top of its low prevalence, DD is often associated with relatively better functioning and a poorer insight which may prevent help seeking behaviour. Methodological constraints and mixed findings further prevent a conclusion from being drawn.

One relevant seminal review of 17 Western studies suggested that DD patients were more likely to be females, married, an immigrant, socially disadvantaged, and have an older age of onset and shorter hospitalisation [11]. However, the included studies dated before the standardisation of DD diagnostic criteria, and therefore lacked appreciation for the different manifestations of the disorder. Other studies suggest a comparable co-morbidity rate with affective disorders and suicidal behaviour between DD and SZ [12, 13], though DD demonstrates a greater susceptibility to physical conditions [14] than SZ [15].

From a functioning perspective however, later studies indicate DD patients to display better social functioning than those with SZ. A longitudinal German study concluded great dissimilarity between DD and paranoid SZ in family history for mental disorders, age of onset, illness course, symptoms, and outcomes [16]. However, only inpatients were included which may have introduced sampling bias with patients who are females and experienced a later onset with more severe symptoms. Another comparative study of DD, paranoid SZ, and non-paranoid SZ in Spain has also indicated DD to be associated with an older age of onset and at index admission, better premorbid adjustment, poorer antipsychotic response, greater likelihood of being married, fewer hospitalisations, and better overall functioning [6].

### Adjusting for age in the exploration of DD vs. SZ differences

Evidence presenting DD and SZ as distinct entities often fails to consider a potentially important confounder – age. Age represents a key prognostic factor in psychosis [17], as older adults are more likely to have established social networks and occupational achievements. Given DD’s older age of onset, comparative studies to other psychoses without adjusting for age may be more inclined to find better premorbid adjustment and functioning in DD.

Indeed, no significant differences in symptom severity, functioning, or neurocognitive performance were found between age-matched DD and SZ patients, except that the former was more likely to be married and have less premorbid schizoid and schizotypal traits [18]. However, another cross-sectional study reports better global functioning in DD than SZ despite age adjustments [7]. With such inconsistent findings, there is a need to further investigate if DD is distinct from SZ in age-matched studies, or whether an extension of the psychosis spectrum is more appropriate. The diagnostic instability of DD further compels the necessity of such research [9], especially given the almost exclusive focus on Western samples thus far. This study aims to investigate the diagnostic stability and the differences in clinical, functional, and neurocognitive outcomes between age-matched DD and SZ cohorts in Hong Kong from Hui et al.’s study [18] at four years.

## Methods

### Study design and participants

Participants were recruited from a population-based, territory-wide Jockey Club Early Psychosis (JCEP) study involving 360 adult patients with first-episode psychosis (FEP), where they were randomized to receive either two or four years of early intervention, or four years of standard care treatment in Hong Kong [19, 20]. Participants were included in the JCEP study if they had received antipsychotic treatment for no longer than 12 months since their first episode; were aged 26–55; were ethnically Chinese and spoke Cantonese; and met the DSM-IV [3] diagnostic criteria for schizophrenia, schizophreniform disorder, schizoaffective disorder, delusional disorder, brief psychotic disorder, psychotic disorder not otherwise specified, or manic episodes with psychotic features. Exclusion criteria include organic brain conditions, substance-induced psychoses, a known history of intellectual disability, and suicidal/violent risks.

**Fig. 1:**
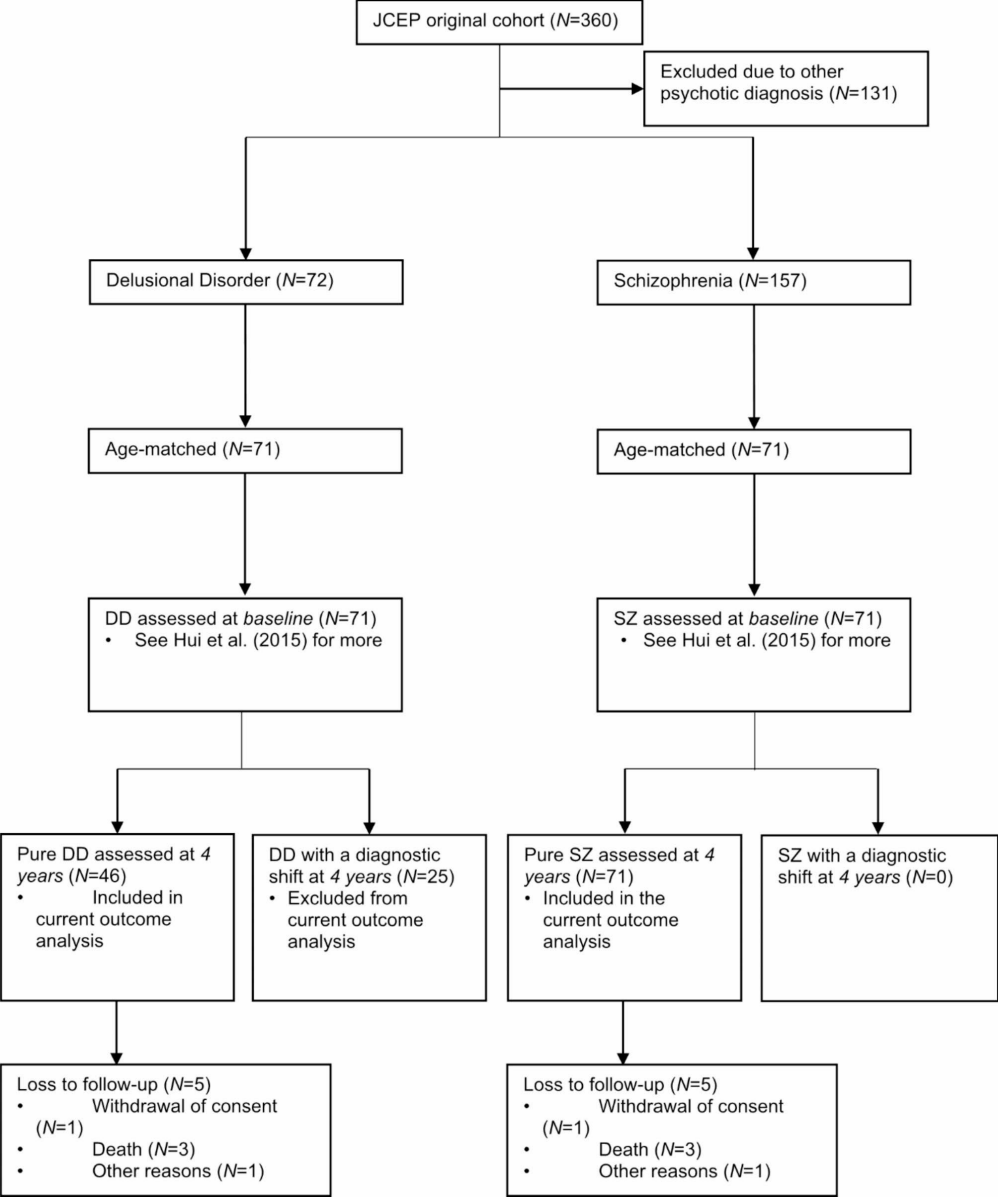
Flow diagram of patients at each phase of the study

As part of this 4-year trial, the baseline characteristics between age-matched patients with DD (*N* = 71) and SZ (*N* = 71) were analyzed in a previous study [18]. The current analysis further examines the four-year outcomes of these DD and SZ patients (Fig. 1). Diagnostic ascertainment was achieved using the validated Chinese version of the Structured Clinical Interview for DSM-IV [21], medical records, and illness history taken from informants and JCEP case workers. Two experienced psychiatrists further confirmed the diagnosis by using the best-estimate consensus approach [22]. Diagnosis of DD and SZ was similarly reassessed at 4 years by psychiatrists.

The ethics of the study were approved by the Institutional Review Board at each study site and was carried out in accordance with Good Clinical Practice as well as the Declaration of Helsinki. All participants provided written informed consent.

### Assessments

Demographic information was sampled from baseline. The following clinical and functioning measures were also evaluated at baseline and at four years.

Symptoms of psychosis were assessed using the Positive and Negative Syndrome Scale (PANSS) [23]. Depressive and mania symptoms were assessed using the Calgary Depression Scale for Schizophrenia (CDSS) [24] and the Young Mania Rating Scale (YMRS) [25].

Insight was measured using the shortened Scale to assess Unawareness of Mental Disorder (SUMD) [26]. Medication side effects and adherence were assessed by the Simpson-Angus Scale (SAS) [27], the Abnormal Involuntary Movement Scale (AIMS) [28], the Barnes Akathisia Rating Scale (BARNS) [29], the Udvalg for Kliniske Undersøgelser (UKU) [30], and the Medication Compliance Questionnaire, a modified Cantonese version of the Medication Adherence Rating Scale [31].

Functioning and quality of life were indicated using the Social Occupational Functioning Assessment Scale (SOFAS) [32], the Role Functioning Scale (RFS) [33], and the 12-item Short Form health survey (SF-12) [34]. Neurocognitive performance was assessed using the Digit Symbol Substitution, Logical Memory, Verbal Fluency and Visual Patterns, and Wisconsin Card Sorting tests.

### Statistical analysis

Analysis was carried out using IBM® SPSS® Version 24.0. Patients with a diagnostic change at four-year were excluded from analysis.

Baseline comparisons between the DD and SZ cohorts were conducted using independent sample t-tests and Mann-Whitney U tests for continuous variables, and Chi-Squared tests for categorical variables. Significant variables (i.e., years of intervention received) were statistically controlled for in subsequent analyses.

Outcome differences were evaluated using binary logistic regression for categorical variables and linear regression for continuous variables. In total, 10 patients (*n =* 5 DD; *n* = 5 SZ) were lost to follow-up at four years due to death (*n* = 6), withdrawal of consent (*n* = 2), or other reasons (*n* = 2) (Fig. 1). Their outcomes were therefore excluded from the four-year comparative analysis. Analysis was repeated to compare pure DD and SZ patients from the JCEP cohort at baseline and at 4-year, without matching for age [20]. To reduce the probability of falsely rejecting a null hypothesis during multiple testing, a false discovery rate (q-value) of 10% with the Benjamini-Hochberg procedure was used [35].

## Results

### Baseline comparisons

There were no statistical baseline differences in age, sex, employment status, years of education, duration of untreated psychosis (DUP), and medication intake between DD and SZ, nor in clinical symptoms or functioning at baseline (Table 1). However, patients with DD received more years of JCEP intervention.

**Table 1:** Baseline comparisons of demographic, treatment, and psychopathology between patients with delusional disorder and schizophrenia

Baseline variables,^†^ mean (SD)		DD (*n* = 46)		SZ (*n* = 71)	Statistics	P-value	
Age (46, 71)		42.5 (7.8)		40.8 (8.7)	*t =* 1.04	0.299	
Male, n (%) (46, 71)		21 (45.7)		30 (42.3)	*X*^2^ = 0.13	0.717	
Employed, n (%) (46, 71)		29 (63.0)		38 (53.5)	*X*^2^ = 1.03^a^	0.309	
Years of education (46, 71)		9.8 (4.2)		10.1 (3.6)	*t =* -0.47	0.636	
Years of JCEP intervention (46, 71)		2.3 (1.6)		1.5 (1.6)	*t =* 2.55	**0.012**	
Duration of untreated psychosis (days) (46, 71)		700.5 (1076.7)		541.9 (1167.0)	*Z* = -1.09	0.276	
Total CPZe, mg/d (45, 69)		166.4 (145.3)		190.6 (136.2)	*t* = -0.90	0.368	
Comorbidity, n (%) (5, 1)					*X*^2^ = 1.20	0.549	
	Affective disorder	2 (40.0)		1 (100.0)			
	Obsessive-compulsive disorder	1 (20.0)		0 (0.0)			
	Other comorbid conditions	2 (40.0)		0 (0.0)			
PANSS (46, 71)							
	Total	50.1 (13.9)		48.1 (13.7)	*t =* 0.74	0.462	
	Positive	10.7 (4.3)		10.0 (4.1)	*t =* 0.93	0.356	
	Negative	9.6 (3.6)		10.5 (5.0)	*t =* -1.07	0.287	
	General psychopathology	25.8 (8.1)		24.0 (7.6)	*t =* 1.21	0.229	
SOFAS^‡^ (46, 71)		56.5 (14.7)		57.6 (11.1)	*t =* -0.41	0.681	

CPZe = chlorpromazine equivalent; DD = delusional disorder; *n =* number; JCEP = Jockey Club Early Psychosis; PANSS = Positive and Negative Syndrome Scale; SD = standard deviation; SOFAS = Social Occupational Functioning Assessment Scale; SZ = schizophrenia.

^†^ Number of available observations for DD and SZ in brackets

^‡^ SOFAS was used to assess the overall social and occupational functioning of an individual on a scale ranging from 1 (grossly impaired) to 100 (excellent functioning)

### Diagnostic stability

Only patients with a pure diagnosis across the four years were included in the outcome analyses (Fig. 1). Patients who were diagnosed with SZ at baseline all remained as such at four years. Amongst the 71 DD patients, 46 (65%) maintained the same diagnosis while 25 (35%) shifted to a SZ diagnosis at four years. There was no statistical difference in the demographics, DUP, or clinical symptoms at baseline between patients *with* and *without* a diagnostic shift (see Additional File 1).

### Clinical outcomes

DD patients had significantly higher PANSS general psychopathology sub-scores at four years (Table 2). No significant differences were found between the two groups in positive, negative, depressive, and mania symptoms. Although there were no differences between the two groups in total antipsychotics intake dosage and side effects, DD patients exhibited better attitude towards medication (Table 3). DD patients also had significantly higher SUMD total and item scores indicating a more impaired insight than SZ patients.

**Table 2:** The four-year symptomatic outcomes of patients with delusional disorder and schizophrenia

Outcomes,^†^ mean (SD)		DD (*n* = 46)	SZ (*n* = 71)	B (95% CI)	P-value
PANSS (41, 66)					
	Total	40.0 (8.8)	38.3 (9.4)	-2.16 (-5.83 to 1.50)	0.245
	Positive	8.6 (2.4)	8.7 (3.7)	-0.01 (-1.31 to 1.29)	0.989
	Negative	9.7 (3.4)	10.1 (3.8)	0.32 (-1.16 to 1.80)	0.670
	General psychopathology	21.7 (5.0)	19.6 (4.2)	-2.47 (-4.26 to -0.68)	**0.007** ^**‡**^
Mood (41, 66)					
	CDSS total	1.6 (2.6)	1.1 (1.8)	-0.63 (-1.49 to 0.23)	0.147
	YMRS total	0.2 (0.5)	0.4 (1.6)	0.33 (-0.20 to 0.86)	0.215

CDSS = Calgary Depression Scale for Schizophrenia; DD = delusional disorder; *n =* number; PANSS = Positive and Negative Syndrome Scale; SD = standard deviation; SZ = schizophrenia; YMRS = Young Mania Rating Scale

^†^ Number of available observations for DD and SZ in brackets

^‡^ Result remains significant after correction by false discovery rate (q-value) of 10% with the Benjamini-Hochberg procedure

**Table 3:** The four-year outcomes on medication side effects and compliance in patients

Outcomes,^†^ mean (SD)		DD (*n* = 46)	SZ (*n* = 71)	B (95% CI)	P-value
Total CPZe, mg/d (35, 60)		204.6 (163.8)	262.1 (243.8)	50.60 (-43.07 to 144.28)	0.286
Side Effects					
	SIMS mean (36, 62)	0.03 (0.1)	0.02 (0.1)	-0.01 (-0.04 to 0.02)	0.482
	BARS global (36, 62)	0.1 (0.4)	0.1 (0.4)	-0.01 (-0.18 to 0.16)	0.877
	AIMS mean (36, 62)	0.0 (0.0)	0.02 (0.1)	0.03 (-0.004 to 0.06)	0.082
	UKU psychic mean (36, 60)	0.1 (0.2)	0.2 (0.3)	0.06 (-0.04 to 0.16)	0.243
	UKU neurological mean (36, 60)	0.03 (0.1)	0.05 (0.1)	0.01 (-0.02 to 0.05)	0.521
	UKU autonomic mean (36, 60)	0.03 (0.1)	0.04 (0.1)	0.01 (-0.03 to 0.05)	0.725
	UKU others (36, 60)	0.02 (0.04)	0.03 (0.1)	0.01 (-0.01 to 0.03)	0.399
Insight SUMD (41, 66)					
	Total	1.7 (0.7)	1.4 (0.5)	-0.36 (-0.60 to -0.13)	**0.003** ^**‡**^
	Awareness of mental disorder	1.8 (0.8)	1.3 (0.6)	-0.50 (-0.76 to -0.24)	**< 0.001** ^**‡**^
	Consequences of mental disorder	1.7 (0.8)	1.3 (0.6)	-0.39 (-0.66 to -0.13)	**0.004** ^**‡**^
	Effects of medication	1.6 (0.7)	1.4 (0.6)	-0.20 (-0.47 to 0.07)	0.152
MCQ – attitudes (34, 50)		2.5 (0.3)	2.3 (0.4)	-0.17 (-0.33 to -0.01)	**0.038**
MCQ – behaviours (34, 50)		3.4 (0.6)	3.5 (0.6)	0.13 (-0.15 to 0.40)	0.360

AIMS = Abnormal Involuntary Movement Scale; BARS = Barnes Akathisia Rating Scale; CPZe = chlorpromazine equivalent; DD = delusional disorder; MCQ = Medication Compliance Questionnaire; *n =* number; SD = standard deviation; SIMS = Simpson Angus Scale; SUMD = Scale to assess Unawareness of Mental Disorder; SZ = schizophrenia; UKU = Udvalg for Kliniske Undersøgelser

^†^ Number of available observations for DD and SZ in brackets

^‡^ Result remains significant after correction by false discovery rate (q-value) of 10% with the Benjamini-Hochberg procedure

### Functioning and neurocognitive outcomes

At four years, DD and SZ patients showed no statistical differences in occupational status, functioning, neurocognitive performance, and quality of life (Table 4).

**Table 4:** Four-year outcomes on functioning, cognitive functioning, and quality of life in patients

Outcomes^†^		DD (n = 46)	SZ (*n* = 71)	B (95% CI)	P-value
Number of relapses*, mean (SD) (41, 66)		0.5 (0.9)	0.7 (1.1)	0.23 (-0.19 to 0.65)	0.280
Social and occupational functioning, mean (SD) (41, 66)					
	Working/studying (n, %)	26 (63.4%)	44 (66.7%)	0.84 (0.37 to 1.93)	0.685
	SOFAS^‡^	59.7 (10.6)	61.1 (8.8)	1.72 (-2.10 to 5.53)	0.375
	RFS^§^ work productivity	4.8 (1.7)	5.0 (1.6)	0.22 (-0.44 to 0.88)	0.508
	RFS^§^ independent living/ self-care	6.1 (0.9)	6.0 (0.7)	-0.02 (-0.35 to 0.30)	0.894
	RFS^§^ immediate social network	5.2 (1.0)	5.4 (1.1)	0.31 (-0.11 to 0.73)	0.145
	RFS^§^ extended social network	4.8 (1.0)	5.0 (1.1)	0.27 (-0.16 to 0.70)	0.212
Cognitive functioning, mean (SD)					
	Logical memory test – immediate (36, 53)	10.1 (4.9)	9.7 (4.9)	-0.19 (-2.33 to 1.95)	0.862
	Logical memory test – delay (36, 53)	7.9 (4.6)	7.2 (4.9)	-0.58 (-2.68 to 1.53)	0.586
	Digit Symbol, adjusted to chronological age (35, 53)	8.3 (4.1)	8.7 (3.5)	0.79 (-0.81 to 2.39)	0.329
	Verbal Fluency – correct response (29, 46)	16.8 (7.1)	16.9 (5.3)	0.31 (-2.59 to 3.21)	0.833
	Digit span – forward (36, 55)	12.0 (2.2)	12.2 (1.9)	0.26 (-0.61 to 1.13)	0.557
	Digit span – backward (36, 55)	7.0 (3.2)	6.5 (3.1)	-0.32 (-1.68 to 1.04)	0.642
	Visual patterns test – correct items (35, 54)	15.8 (6.8)	16.4 (5.3)	1.12 (-1.46 to 3.70)	0.392
	WCST, perseveration error (35, 54)	4.4 (4.5)	7.2 (8.7)	2.67 (-0.60 to 5.94)	0.108
Quality of life, mean (SD) (36, 54)					
	SF-12 – mental component	59.2 (27.4)	65.6 (23.1)	8.40 (-2.35 to 19.16)	0.124
	SF-12 – physical component	64.7 (30.5)	66.8 (27.4)	3.93 (-8.54 to 16.39)	0.533

DD = delusional disorder; *n =* number; RFS = Role Functioning Scale; SD = standard deviation; SF-12 = 12-item short-form health survey.; SOFAS = Social Occupational Functioning Assessment Scale; SZ = schizophrenia; WCST = Wisconsin Card Sorting Test

^†^ Number of available observations for DD and SZ in brackets

* Relapse was defined as a CGI score greater than or equal to 3 after a remission period of at least 3 months

^‡^ SOFAS was used to assess the overall social and occupational functioning of an individual on a scale ranging from 1 (grossly impaired) to 100 (excellent functioning)

^§^ RFS was used to assess the role functioning of an individual on a seven-point scale for four constituent components: work productivity, independent living and self-care, immediate social network relationships, and extended social network relationships. Scores ranged from 1 (severe impairment) to 7 (excellent and optimal functioning)

Overall, aside from attitude towards medication, group differences remained significant for general psychopathology and insight in mental disorder after corrections using the Benjamini-Hochberg procedure [35].

In order to evaluate whether matched and non-matched samples would yield differential outcomes, analyses were repeated for all DD (*n* = 72) and SZ (*n* = 157) patients from the JCEP cohort [20] without matching for age (see Additional File 2). As expected, DD and SZ cohorts showed significant baseline differences in age and employment status. Attitude towards medication did not differ in non-matched patients after controlling for employment status. However, differences relating to general psychopathology and insight remained significant.

## Discussion

Previous studies comparing the outcomes of DD and SZ patients have either only included inpatients or neglected to match for a crucial prognostic factor in schizophrenia – age. Continuing from an earlier cross-sectional study on Chinese patients [18], this was the first prospective study investigating the diagnostic stability and clinical, functional, and neurocognitive differences between age-matched DD and SZ Chinese cohorts at four years.

Notably, few studies have explored DD and SZ in non-Western populations despite potential cultural differences. For instance, familial support may be more accessible in Hong Kong considering that 87% of FEP patients live in a family household [36]. Such support may be especially important given that 42% of patients require medication reminders by family members or carers [37], Indeed, only 26% of patients with SZ demonstrate non-adherence to medication in Hong Kong [38] in comparison to 50% in Western countries [39]. However, the stigmatisation of psychosis in Hong Kong [40] may lead to poorer social and occupational functioning due to exclusion from employers [41]. Other differences include local substance abuse, as the prevalence in Hong Kong [42] is less than one-tenth of Denmark [43]. Substance abuse not only poses a clinical challenge for diagnostic ascertainment, but is also associated with poorer functioning [44]. Therefore, the current study provides a unique insight into DD and SZ beyond a Western perspective in Hong Kong.

### Diagnostic stability

In line with previous evidence of the diagnostic instability [9], a large proportion of our DD patients (35%) underwent a diagnostic reassignment to SZ by the fourth year. The diagnostic stability of SZ may be related to its stringent diagnostic requirement, such that symptoms should be present for at least six months while no such specification exists for DD. Alternatively, the distinction between DD and SZ may be arbitrary in reality – the diagnostic conversion may reflect a natural course of illness rather than an actual diagnostic change [45, 46].

### Clinical outcomes

Our study showed that pure DD is associated with more general psychopathology symptoms compared to SZ, even after accounting for the poorer insight reported in DD. A post hoc analysis of PANSS general psychopathology found significant results even after removing item G12 (insight; *b* = -1.632, *p* = .037, *CI* -3.160 to -0.104). The significant difference may therefore be driven by a collective range of symptoms such as depression, anxiety, and other behavioural symptoms. Our findings may thus pave the path for an enhanced characterisation of DD that moves beyond the content of delusions to the underlying psychopathological structure of the disorder.

### Medication side effects, compliance, and insight

Our findings revealed that DD patients had more impaired insight, particularly in their awareness of the mental illness and its consequences. With delusions being its defining symptom, it is perhaps unsurprising that DD patients were unable to differentiate between the creations of their own mind and reality, thus failing to recognise their mental illness. There may also be an underlying tendency for DD patients to make erroneous inferences with high conviction. Greater over-confidence in judgement has been associated with the presence of delusions in psychotic disorders [47]. Not only may the lack of insight hinder help-seeking and treatment adherence, but it may also impede development of therapeutic relationships [48].

Despite no difference in side effects, DD patients displayed a better attitude towards medication. These findings are rather contentious considering that both side effects and insight have been shown to predict medication attitude [49]. However, studies of medication attitude often neglect to consider other moderating factors (e.g., cognitive impairments) [50] and its multidimensionality. This includes the dimensions of necessity and concerns about medication, of which only the former relates to illness awareness [51]. Although speculative, the smaller dosage of medication taken by the DD cohort (Table 3) may have also influenced their medication attitudes in the current study.

Furthermore, the dimensions of SUMD may be differentially related to medication attitude and therefore should be considered individually. An earlier study suggested that amongst the three aspects of insight, only awareness of medication impact was important for medication attitude [52]. Given that DD patients only demonstrated an impaired insight towards the presence of their mental illness and its consequences, that they also express better attitudes to medication may not be as contradictory as first assumed. Regardless, the difference in medication attitude failed to remain significant after a false discovery rate correction which suggests that findings should be interpreted with caution.

### Functioning, neurocognitive performance, and quality of life

At four years, social and occupational functioning were comparable between the cohorts. Matching the two groups for age, sex, and years of education may account for why the present findings contradict previous studies [11]. In a secondary analysis that was conducted as part of our baseline study [18], level of functioning *did* differ between the two groups when age-matching was omitted.

Neurocognitive functions were also found to be similar between the DD and SZ groups in our study. This finding resonates with that of the baseline study [18] and other relevant studies. For example, an earlier age-matched comparison of DD and SZ patients found comparable attention level, verbal and motor skills, cognitive flexibility, abstract thinking, memory, and psychomotricity [53]. A similar study that matched for age and years of education found no differences between DD and paranoid SZ patients in verbal learning, general memory, and sustained attention [54]. However, DD patients displayed better overall functioning than SZ patients when not matched for age [16]. The discrepancy in findings may be attributed to the potentially confounding effects of age on functioning, which may have resulted in the illusion that DD is associated with better functioning when it is not controlled for. Another interpretation is that age may have a stronger bearing on neurocognitive functions in DD relative SZ.

### Limitations

This study had the following limitations. Firstly, this prospective follow-up study was conducted as a part of an intervention study [20], with a predetermined duration of treatment that can only be statistically controlled for. Secondly, only FEP patients aged 26–55 were included; the scarcity of adolescent cases made it difficult to recruit a sizable younger sample. The finding that DD and SZ are not as different as previously thought may therefore not apply to the adolescent population.

While it may have been preferable to only compare patients with more acute symptoms at study entry, patients were often only referred to the JCEP early intervention service four months after treatment. As a result, their clinical presentation may have already reduced in severity at study entry. In addition, statistical power may have been undermined by the removal of patients with a diagnostic shift at 4 years. Therefore, findings should be replicated in a larger sample with more acute presentations to provide further support for the lack of differences between DD and SZ.

Although age-matching removes a critical confounding variable, it also simultaneously hinders the exploration of the effects of age on outcomes. Age could be indicative of the impact of long-term illness or the cumulated use of antipsychotics on brain tissue atrophy over time [55]. Nevertheless, repeating the analysis without matching for age yielded similar results except that medication attitude failed to reach significance. This further reinforces the notion that DD and SZ may truly share similar longitudinal outcomes.

## Conclusion

By comparing the four-year outcomes of age-matched DD and SZ patients, we found that the main differences laid in clinical factors. DD patients experienced more general psychopathology symptoms with a poorer illness insight and a better medication attitude. However, the two groups were largely similar in terms of social and occupational functioning, neurocognitive outcomes, and quality of life. Therefore, the two cohorts may not be as distinct as previously thought when the effects of potential confounders such as age, sex, and years of education are minimised. We believe that these findings will be useful in the characterisation of DD as a continuum of SZ, as well as in the advocacy for a dimensional approach to psychosis spectrum disorders.

### Electronic supplementary material

Below is the link to the electronic supplementary material.

**Supplementary Material 1.** BMC_additional file 1. Title: “Supplementary data on diagnostic stability”.. Contains a table to show the baseline comparison of demographic and psychopathology variables between delusional disorder patients with and without a diagnostic shift to schizophrenia after four years.

**Supplementary Material 2.** BMC_additional file 2. Title: “Supplementary data on non-matched comparative analysis”.. The file provides detailed descriptions of the non-matched comparative analysis among 72 patients with delusional disorder and 157 patients with schizophrenia from the original cohort that has *not* been matched for age. Four tables are included to show the baseline and 4-year outcome comparisons between patients with delusional disorders and schizophrenia.

## Data Availability

The data that support the findings of this study are available from the corresponding author, CLMH, upon reasonable request.

## Abbreviations

AIMS: Abnormal Involuntary Movement Scale
BARNS: Barnes Akathisia Rating Scale
CDSS: Calgary Depression Scale for Schizophrenia
DD: Delusional Disorder
DUP: Duration of Untreated Psychosis
EI: Early Intervention
FEP: First Episode Psychosis
JCEP: Jockey Club Early Psychosis
PANSS: Positive and Negative Syndrome Scale
RFS: Role Functioning Scale
SAS: Simpson-Angus Scale
SF-12: 12-item Short Form health survey
SOFAS: Social Occupational Functioning Assessment Scale
SUMD: Scale to assess Unawareness of Mental Disorder
SZ: Schizophrenia
UKU: Udvalg for Kliniske Unders?gelser
YMRS: Young Mania Rating Scale
