# Supplementary material for: Functional and clinical outcomes of delusional disorder and schizophrenia patients after first episode psychosis: a 4-year follow-up study
Source: BMC Psychiatry. 2023 Sep 18;23:676. doi: 10.1186/s12888-023-05175-z (PMC10506281; doi:10.1186/s12888-023-05175-z)
Supplement: Supplementary file 1 — Supplementary Material 1. BMC_additional file 1. Title: “Supplementary data on diagnostic stability”.. Contains a table to show the baseline comparison of demographic and psychopathology variables between delusional disorder patients with and without a diagnostic shift to schizophrenia after four years. [file 12888_2023_5175_MOESM1_ESM.docx]

**Additional file 1: Supplementary data on diagnostic stability**

The supplementary table 1 below reveals that there were no statistical differences in the demographics, duration of untreated psychosis (DUP), and clinical symptoms at baseline between DD patients *with* (*n*=25) and *without* a diagnostic shift to schizophrenia (*n*=46).

**Supplementary table 1.** Baseline comparison of demographic and psychopathology variables between delusional disorder patients with and without a diagnostic shift to schizophrenia.

| Variables,^†^ mean (SD) | | Diagnostic shift (*n*=25) | No diagnostic shift (*n*=46) | Statistics | P-value |
| --- | --- | --- | --- | --- | --- |
| Age (25, 46) | | 40.4 (9.1) | 42.5 (7.8) | *t* = -1.02 | 0.311 |
| Male, n (%) (25, 46) | | 10 (40.0) | 21 (45.7) | *X*^2^ = 0.21 | 0.646 |
| Employed, n (%) (25, 46) | | 12 (48.0) | 29 (63.0) | *X*^2^ = 1.50 | 0.220 |
| Years of education (25, 46) | | 9.6 (4.1) | 9.8 (4.2) | *t* = -0.14 | 0.891 |
| Duration of untreated psychosis (days) (25, 46) | | 759.0 (1707.9) | 700.5 (1076.7) | *t* = 0.18 | 0.860 |
| Total CPZe, mg/d (23, 45) | | 122.3 (83.9) | 166.4 (145.3) | *t* = -1.34 | 0.184 |
| PANSS (25, 46) | |  |  |  |  |
|  | Total | 51.5 (14.8) | 50.1 (13.9) | *t* = 0.41 | 0.682 |
|  | Positive | 10.3 (4.3) | 10.7 (4.3) | *t* = -0.41 | 0.682 |
|  | Negative | 11.1 (4.9) | 9.6 (3.6) | *t* = 1.47 | 0.145 |
|  | General psychopathology | 26.3 (8.6) | 25.8 (8.1) | *t* = 0.27 | 0.785 |

CPZe=chlorpromazine equivalent; DD=delusional disorder; *n=*number; PANSS=Positive and Negative Syndrome Scale; SD=standard deviation; SZ=schizophrenia.

^†^ Number of available observations for DD and SZ in brackets.
