# Supplementary material for: Functional and clinical outcomes of delusional disorder and schizophrenia patients after first episode psychosis: a 4-year follow-up study
Source: BMC Psychiatry. 2023 Sep 18;23:676. doi: 10.1186/s12888-023-05175-z (PMC10506281; doi:10.1186/s12888-023-05175-z)
Supplement: Supplementary file 2 — Supplementary Material 2. BMC_additional file 2. Title: “Supplementary data on non-matched comparative analysis”.. The file provides detailed descriptions of the non-matched comparative analysis among 72 patients with delusional disorder and 157 patients with schizophrenia from the original cohort that has not been matched for age. Four tables are included to show the baseline and 4-year outcome comparisons between patients with delusional disorders and schizophrenia. [file 12888_2023_5175_MOESM2_ESM.docx]

**Additional file 2: Supplementary data on non-matched comparative analysis**

The outcomes of patients with DD (*n*=72) were compared to those with SZ (*n*=157) without having matched for age. Twenty-six DD patients were excluded from the analysis due to having had a diagnosis shift by 4 years. Only age and employment status significantly differed between DD and SZ. Gender ratio, DUP, years of education, and years of intervention received did not differ at baseline. Neither was there a baseline difference in total chlorpromazine equivalent dosage, comorbidity, PANSS total or subscale scores, or SOFAS (**Supplementary table 2** below). Thus, employment status was controlled for in subsequent analyses.

Relative to findings in the age-matched sample, all significant group differences at 4 years remained significant (**Supplementary table 3**) with the exception of attitude towards medication which did not significantly vary between DD and SZ (**Supplementary table 4**). Patients with DD continued to report higher PANSS general psychopathology than those with SZ. DD patients also expressed higher SUMD total scores, as well as higher SUMD item scores for awareness of mental disorder and awareness of the consequences of mental disorder. There were no significant differences between the two groups in social and occupational functioning, cognitive functioning, and quality of life (**Supplementary table 5**). All significant findings remained significant after applying a false discovery rate of 10% with the Benjamini-Hochberg procedure.

**Supplementary table 2**. Baseline comparisons of demographic, treatment, and psychopathology variables between non-matched patients with delusional disorder and schizophrenia.

| Baseline variables,^†^ mean (SD) | | DD (*n*=72) | SZ (*n*=157) | Statistics | P-value |
| --- | --- | --- | --- | --- | --- |
| Age (46, 157) | | 42.5 (7.8) | 37.6 (7.9) | *t* = 3.69 | **<0.001** |
| Male, n (%) (46, 157) | | 21 (45.7) | 75 (47.8) | *X*^2^ = 0.06 | 0.800 |
| Employed, n (%) (46, 157) | | 29 (63.0) | 69 (43.9) | *X*^2^ = 5.20 | **0.023** |
| Years of education (46, 157) | | 9.8 (4.2) | 10.7 (3.6) | *t* = -1.51 | 0.132 |
| Years of JCEP intervention (46, 157) | | 2.3 (1.6) | 1.9 (1.6) | *t* = 1.39 | 0.165 |
| Duration of untreated psychosis (days) (46, 157) | | 700.5 (1076.7) | 709.7 (1219.5) | *t* = -0.05 | 0.964 |
| Total CPZe, mg/d (45, 154) | | 166.4 (145.3) | 183.8 (123.2) | *t* = -0.89 | 0.373 |
| Comorbidity, n (%) (5, 6) | |  |  | *X*^2^ = 3.47 | 0.482 |
|  | Affective disorder | 2 (40.0) | 3 (50.0) |  |  |
|  | Substance Abuse | 0 (0.0) | 1 (16.7) |  |  |
|  | Obsessive-compulsive disorder | 1 (20.0) | 0 (0.0) |  |  |
|  | Other comorbid conditions | 2 (40.0) | 1 (16.7) |  |  |
|  | More than one comorbid condition | 0 (0.0) | 1 (16.7) |  |  |
| PANSS (46, 157) | |  |  |  |  |
|  | Total | 50.1 (13.9) | 47.4 (13.1) | *t* = 1.19 | 0.234 |
|  | Positive | 10.7 (4.3) | 9.8 (4.1) | *t* = 1.37 | 0.173 |
|  | Negative | 9.6 (3.6) | 10.6 (4.7) | *t* = -1.38 | 0.170 |
|  | General psychopathology | 25.8 (8.1) | 23.5 (7.3) | *t* = 1.84 | 0.068 |
| SOFAS^‡^ (46, 157) | | 56.5 (14.7) | 57.1 (12.2) | *t* = -0.260 | 0.795 |

CPZe=chlorpromazine equivalent; DD=delusional disorder; *n=*number; PANSS=Positive and Negative Syndrome Scale; SD=standard deviation; SOFAS= Social Occupational Functioning Assessment Scale SZ=schizophrenia.

^†^ Number of available observations for DD and SZ in brackets.
^‡^ SOFAS was used to assess the overall social and occupational functioning of an individual on a scale ranging from 1 (grossly impaired) to 100 (excellent functioning).

**Supplementary table 3.** The four-year symptomatic outcomes of non-matched patients with delusional disorder and schizophrenia.

| Outcomes,^†^ mean (SD) | | DD (*n*=46) | SZ (*n*=157) | B (95% CI) | P-value |
| --- | --- | --- | --- | --- | --- |
| PANSS (41, 144) | | | | | |
|  | Total | 40.0 (8.8) | 38.2 (8.3) | -1.91 (-4.90 to 1.07) | 0.207 |
|  | Positive | 8.6 (2.4) | 8.7 (3.3) | 0.01 (-1.09 to 1.11) | 0.985 |
|  | Negative | 9.7 (3.4) | 9.9 (3.5) | 0.18 (-1.04 to 1.41) | 0.769 |
|  | General psychopathology | 21.7 (5.0) | 19.6 (4.0) | -2.11 (-3.60 to -0.61) | **0.006^‡^** |
| Mood (41, 144) | | | | | |
|  | CDSS total | 1.6 (2.6) | 0.9 (1.7) | -0.61 (-1.29 to 0.07) | 0.080 |
|  | YMRS total | 0.2 (0.5) | 0.6 (2.3) | 0.41 (-0.32 to 1.13) | 0.268 |

CDSS=Calgary Depression Scale for Schizophrenia; DD=delusional disorder; *n=*number; PANSS=Positive and Negative Syndrome Scale; SD=standard deviation; SZ=schizophrenia; YMRS=Young Mania Rating Scale.

^†^ Number of available observations for DD and SZ in brackets.

^‡^ Result remains significant after correction by false discovery rate (q-value) of 10% with the Benjamini-Hochberg procedure.

**Supplementary table 4.** The four-year outcomes in side effects, insight, and medication compliance of non-matched patients with delusional disorder and schizophrenia.

| Outcomes,^†^ mean (SD) | | DD (*n*=46) | SZ (*n*=157) | B (95% CI) | P-value |
| --- | --- | --- | --- | --- | --- |
| Total CPZe, mg/d (35, 135) | | 204.6 (163.8) | 242.9 (214.2) | 27.86 (-47.71 to 103.43) | 0.468 |
| Side Effects | | | | | |
|  | SIMS mean (36, 139) | 0.03 (0.1) | 0.03 (0.1) | 0.002 (-0.04 to 0.04) | 0.909 |
|  | BARS global (36, 139) | 0.1 (0.4) | 0.1 (0.4) | -0.04 (-0.19 to 0.10) | 0.548 |
|  | AIMS mean (36, 139) | <.01 (<.01) | 0.03 (0.1) | 0.02 (-0.01 to 0.06) | 0.191 |
|  | UKU psychic mean (36, 137) | 0.1 (0.2) | 0.2 (0.3) | 0.05 (-0.04 to 0.15) | 0.257 |
|  | UKU neurological mean (36, 137) | 0.03 (0.1) | 0.05 (0.1) | 0.01 (-0.03 to 0.05) | 0.692 |
|  | UKU autonomic mean (36, 137) | 0.03 (0.1) | 0.03 (0.1) | 0.001 (-0.03 to 0.03) | 0.962 |
|  | UKU others (36, 137) | 0.02 (0.04) | 0.03 (0.1) | 0.01 (-0.01 to 0.03) | 0.422 |
| Insight SUMD (41,144) | | | | | |
|  | Total | 1.7 (0.7) | 1.4 (0.5) | -0.33 (-0.53 to -0.12) | **0.002^‡^** |
|  | Awareness of mental disorder | 1.8 (0.8) | 1.4 (0.6) | -0.44 (-0.66 to -0.21) | **<0.001^‡^** |
|  | Consequences of mental disorder | 1.7 (0.8) | 1.4 (0.6) | -0.35 (-0.58 to -0.13) | **0.002^‡^** |
|  | Effects of medication | 1.6 (0.7) | 1.4 (0.6) | -0.19 (-0.41 to 0.04) | 0.104 |
| MCQ – attitudes (34, 113) | | 2.5 (0.3) | 2.3 (0.4) | -0.13 (-0.28 to 0.03) | 0.110 |
| MCQ – behaviours (34, 113) | | 3.4 (0.6) | 3.5 (0.5) | 0.04 (-0.17 to 0.26) | 0.681 |

*n=*number; SD=standard deviation; DD=delusional disorder; SZ=schizophrenia; CPZe=chlorpromazine equivalent; SIMS=Simpson Angus Scale; BARS=Barnes Akathisia Rating Scale; AIMS=Abnormal Involuntary Movement Scale; UKU= Udvalg for Kliniske Undersøgelser; SUMD=Scale to assess Unawareness of Mental Disorder; MCQ=Medication Compliance Questionnaire.
^†^ Number of available observations for DD and SZ in brackets.

^‡^ Result remains significant after correction by false discovery rate (q-value) of 10% with the Benjamini-Hochberg procedure.

**Supplementary table 5.** The four-year outcomes on functioning, cognitive functioning, and quality of life of non-matched patients with delusional disorder and schizophrenia.

| Outcomes^†^ | | DD (*n*=46) | SZ (*n*=157) | B (95% CI) | P-value |
| --- | --- | --- | --- | --- | --- |
| Number of relapses*, mean (SD) (44, 154) | | 0.5 (0.9) | 0.7 (1.2) | 0.19 (0.20 to 0.58) | 0.330 |
| Social and occupational functioning, mean (SD) (41, 144) | | | | | |
|  | Working/studying (n, %) | 26 (63.4%) | 44 (66.7%) | 0.30 (0.30 to 1.46) | 0.302 |
|  | SOFAS^‡^ | 59.7 (10.6) | 60.3 (9.2) | 1.63 (-1.64 to 4.89) | 0.327 |
|  | RFS^§^ work productivity | 4.8 (1.7) | 5.0 (1.6) | 0.07 (-0.36 to 0.22) | 0.633 |
|  | RFS^§^ independent living/ self-care | 6.1 (0.9) | 5.9 (0.8) | -0.02 (-0.35 to 0.30) | 0.894 |
|  | RFS^§^ immediate social network | 5.2 (1.0) | 5.4 (1.1) | 0.26 (-0.11 to 0.63) | 0.160 |
|  | RFS^§^ extended social network | 4.8 (1.0) | 5.0 (1.2) | 0.18 (-0.22 to 0.58) | 0.374 |
| Cognitive functioning, mean (SD) | | | | | |
|  | Logical memory test – immediate (36, 119) | 10.1 (4.9) | 10.0 (4.8) | -0.19 (-1.68 to 2.06) | 0.839 |
|  | Logical memory test – delay (36, 119) | 7.9 (4.6) | 7.5 (4.9) | -0.19 (-2.07 to 1.70) | 0.843 |
|  | Digit Symbol, adjusted to chronological age (35, 118) | 8.3 (4.1) | 8.6 (3.2) | 0.27 (-1.07 to 1.61) | 0.694 |
|  | Verbal Fluency – correct response (29, 106) | 16.8 (7.1) | 16.8 (5.5) | 0.04 (-2.44 to 2.51) | 0.978 |
|  | Digit span – forward (36, 121) | 12.0 (2.2) | 12.1 (2.0) | 0.12 (-0.67 to 0.91) | 0.769 |
|  | Digit span – backward (36, 121) | 7.0 (3.2) | 6.5 (3.0) | -0.51 (-1.70 to 0.67) | 0.393 |
|  | Visual patterns test – correct items (35, 119) | 15.8 (6.8) | 17.6 (6.0) | 1.70 (-0.71 to 4.10) | 0.166 |
|  | WCST, perseveration error (35, 120) | 4.4 (4.5) | 5.9 (7.1) | 2.00 (-0.55 to 4.56) | 0.124 |
| Quality of life, mean (SD) (36, 54) | | | | | |
|  | SF-12 – mental component | 59.2 (27.4) | 66.0 (22.1) | 6.73 (-2.35 to 15.81) | 0.145 |
|  | SF-12 – physical component | 64.7 (30.5) | 70.3 (24.0) | 5.68 (-4.28 to 15.63) | 0.261 |

DD=delusional disorder; *n=*number; SD=standard deviation; SF-12=12-item short-form health survey; SOFAS=Social Occupational Functioning Assessment Scale; RFS=Role Functioning Scale; SZ=schizophrenia; WCST=Wisconsin Card Sorting Test.
^†^ Number of available observations for DD and SZ in brackets.

* Relapse was defined as a CGI score greater than or equal to 3 after a remission period of at least 3 months.
^‡^ SOFAS was used to assess the overall social and occupational functioning of an individual on a scale ranging from 1 (grossly impaired) to 100 (excellent functioning).
^§^ RFS was used to assess the role functioning of an individual on a seven-point scale for four constituent components: work productivity, independent living and self-care, immediate social network relationships, and extended social network relationships. Scores ranged from 1 (severe impairment) to 7 (excellent and optimal functioning).
